# Seasonal variation in preference dictates space use in an invasive generalist

**DOI:** 10.1371/journal.pone.0199078

**Published:** 2018-07-20

**Authors:** Kelsey E. Paolini, Bronson K. Strickland, Jessica L. Tegt, Kurt C. VerCauteren, Garrett M. Street

**Affiliations:** 1 Department of Wildlife, Fisheries, and Aquaculture, Mississippi State University, Starkville, Mississippi, United States of America; 2 United States Department of Agriculture, National Wildlife Research Center, Fort Collins, Colorado, United States of America; Hungarian Academy of Sciences, HUNGARY

## Abstract

**Background:**

The spatiotemporal distribution of resources is a critical component of realized animal distributions. In agricultural landscapes, space use by generalist consumers is influenced by ephemeral resource availability that may produce behavioral differences across agricultural seasons, resulting in economic and production consequences and increased human-wildlife conflict. Our objective was to assess changes in habitat selection across seasons in an invasive generalist omnivore (feral pigs, *Sus scrofa*). Hypothesizing that pig space use is primarily driven by forage availability, we predicted strong selection for the most nutritionally beneficial crops and resource types as agricultural seasons progressed. We deployed GPS collars on 13 adult feral pigs in the Mississippi Alluvial Valley to study resource selection in a fragmented agricultural landscape. We estimated resource selection using mixed-effect logistic regression to assess variation in selection across planting, growing, harvest, and fallow seasons.

**Results:**

We found that feral pigs varied resource selection across seasons, particularly for corn (*Zea mais*). We also detected seasonal dependencies in proportional coverage on the net probability of selection of a land unit (e.g., selection was generally strongest for locations composed of both agricultural and natural habitat), resulting in marked variation in predicted space use among agricultural seasons.

**Conclusions:**

These findings indicate behavioral changes in selection across agricultural seasons are driven by complex interactions between the availabilities of temporally dynamic resources and temporally static natural cover. Temporal variations in resource selection trends indicate seasonal responses to crop phenology which suggests a season-specific habitat functional response.

## Introduction

The distribution of resources across a landscape is a primary factor governing animal space use patterns. Spatial variation in the distribution of resources is reflected through animal distributions and local densities [1]; however, resource distributions are not only spatially variable, but temporally as well, with resource quality and availability often tracking seasonal changes in biotic and abiotic environmental conditions. Many animals alter their space use seasonally corresponding to changes in forage availability [2,3], predation risk [4], and abiotic environmental conditions [4–6]. An understanding of the drivers of animal space use, and prediction of animal distributions resulting from these drivers, requires description of the contribution of landscape characteristics to animal ecology across both space and time.

Studying spatiotemporal dynamics of animal distributions is particularly appropriate for agricultural systems where ephemeral resources are present across distinct temporal periods corresponding to planting, growing, and harvesting of crops. A hyperabundance of food resources attracts primary consumers, but differences in crop phenologies vary the quantity, digestibility, and nutritional value of crop types throughout the year [7]. In agricultural landscapes, to maximize survivorship, generalist consumers can thrive on seasonally available crops in the planting and growing seasons while increasing population size, and switch consumption largely to naturally occurring resources in the fallow season (i.e., winter; [8]). For example, white-tailed deer (*Odocoileus virginianus*) primarily consume crops in the summer (e.g., soybeans, *Glycine max*), and the amount of consumption depends not only on resource availability in spring and summer, but also quality and abundance of natural forage consumed throughout the winter in adjoining forested habitat [9]. Clearly, agricultural resources can provide critical nutrition to consumers throughout the year, but are of particular importance coming out of the winter season when body condition tends to be lowest (e.g., [10]).

If consumers exhibit distinct seasonal preferences for forage type [8,9,11,12], we should expect to observe distinct seasonal differences in selection for forage types estimated from models of consumer distributions (i.e., Species Distribution Models, SDMs; [13]). Conceptually, this is analogous to a habitat functional response whereby animals change the magnitude and/or direction of selection for a resource in response to changes in its availability [14]. However, in an agricultural landscape this is not only driven by crop availability as described above, but also by crop phenology as it influences quality and digestibility [7], and how it changes the way an animal interacts with the plant itself. Consider for example the seasonal value of corn (*Zea mais*). At planting and during the early growing season, corn is easily apprehended as kernels or seedlings and has high nutritional value to primary consumers [7,12,15]. During plant growth corn becomes less digestible; however, the height and density of corn in most corn fields are such that the crop may provide shelter from predators and the elements. Consumers may still exhibit strong preferences for corn during growth stages and agricultural periods where the quality and accessibility of forage are lower because the driver of resource use has shifted toward non-foraging benefits. However, at kernel maturation corn serves as both shelter and a consumable resource meaning that in agricultural landscapes, organisms may be strongly driven by how the organism perceives and uses a resource to fulfill multiple needs.

Here we investigate the implications of potential changes in crop/consumer interactions across agricultural seasons in an invasive generalist consumer, feral pigs (wild pigs, feral swine, wild hogs, etc.; *Sus scrofa*; [16]). Feral pigs are well-adapted to surviving across a wide array of environmental conditions and have dramatically expanded their ranges in the United States [17–19]. Pigs are known to modify their spatial distributions in response to ephemeral food resources [11,20,21]. The dietary plasticity exhibited by feral pigs classifies pigs as opportunistic omnivores. In agroecosystems, feral pigs largely consume plant material (e.g., corn) when available [12,21]. When crop resource availability diminishes, or other natural foods become seasonally available feral pigs may increase foraging effort for mast such as acorns (*Quercus* spp.) and beechnuts (*Fagus sylvatica*), suggesting that a seasonal switch in dietary preferences is the likely driver of variation in pig spatial distributions across periods of agricultural phenology and activities [12]. However, pigs also rely on cover and water to thermoregulate [22], which could evoke a switch in how they interact with different crops as described earlier. This could drastically modify their seasonal space use trends due to season-specific ecological drivers of preference and behavior in agricultural landscapes.

Our objective was to characterize space use by feral pigs as a function of seasonal changes in resource availability and quality within a fragmented, agricultural landscape. We defined 4 discrete temporal extents based on agricultural activity and crop phenology, analogous to agricultural “seasons” (planting, growing, harvest, and fallow), and fit mixed-effects models of habitat selection to feral pigs within each season. Hypothesizing that feral pigs are energy maximizing foragers whose space use is primarily driven by availability of high quality resources [23,24], we predicted strong selection for the most nutritionally beneficial crops and resource types as agricultural seasons progressed. Specifically, we predicted strong positive selection for (1) corn during the planting season; (2) other primary crops such as soybean and rice (*Oryza sativa*) in the growing and harvest seasons as the corn plant becomes less digestible through maturation and the harvest; (3) bottomland hardwoods and natural wetlands that provide food items such as hard mast (e.g., acorns; [25,26]) during the fallow season; and 4) strong negative selection for increasing distance to water because pigs are reliant on water and canopy cover to facilitate thermoregulation in warmer months [22]. We then characterized the change in selection for different crop types between and within seasons with respect to changes in resource availability. Finally, we projected our models across the landscape to create season-specific predictions of the expected utilization distribution.

## Methods

### Study site

**Fig 1:**
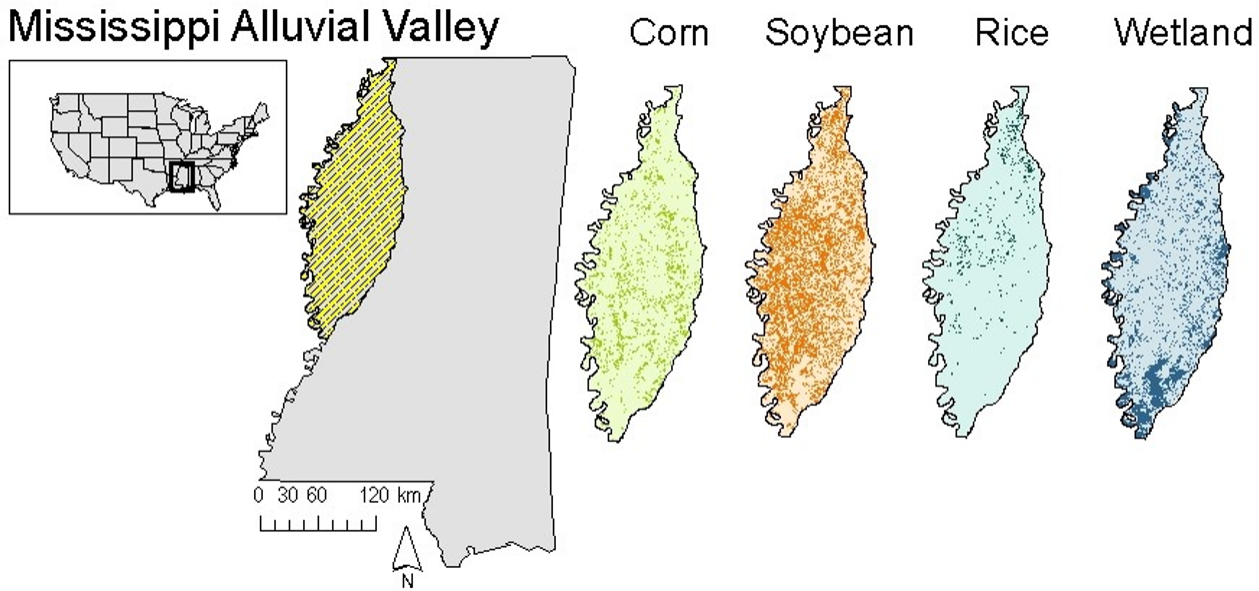
Study site. Location of study site within the northwestern region of the state of Mississippi, USA. The cross-hatched area delineates the Mississippi Alluvial Valley. Sub-maps are rasters of availability of specific land-cover types (dark areas represent greater availability).

We conducted our study in the Lower Mississippi Alluvial Valley (LMAV) located in the northwestern region of Mississippi (Fig 1). The LMAV averages 145 cm of precipitation annually and is characterized by a humid subtropical environment [27]. Bottomland hardwood forests within the LMAV surround the floodplains of the Mississippi River and are commonly composed of willow oak (*Quercus phellos*), water oak (*Quercus nigra*), and green ash (*Fraxinus pennsylvanica*; [27]). Bottomland hardwoods were historically fragmented to permit active agriculture in the rich fertile soils [28]. Within our study region, some of the main agricultural crops included corn (12%), rice (4%) and soybeans (38%), which were interspersed with a mosaic of wetlands (23%; Fig 1; S1 Table).

Bottomland hardwoods and seasonal crop availabilities accommodate both thermoregulatory and anti-predatory cover for feral pigs. Although adult feral pigs do not have natural predators within the LMAV, humans are a consistent source of predation risk. Feral pigs are classified as a nuisance species in Mississippi and can be hunted year-round [29], which increases the value of consistent cover sources.

### Data collection and management

We captured 16 adult feral pigs using baited corral traps in the Mississippi Alluvial Valley (approximately 33° 31' 34.32" N 90° 40' 38.28" W) from November 2015 through May 2015. Trapping locations were opportunistic in the northern LMAV and based on landowner approval. To anesthetize pigs for the collaring procedure, we administered a mixture of Telazol (100 mg/mL when reconstituted), Ketamine (100 mg/mL), and Xylazine HCl (100 mg/mL; TKX). We fitted individuals with Iridium Global Positioning System (GPS) collars (LOTEK Engineering Ltd., Newmarket, Canada (n = 3); Vectronic Aerospace GmbH Berlin, Germany (n = 13)). We collected data through January 2017 and subsampled locations at 2-hour intervals throughout collar deployment. All animal procedures and protocols were strictly followed and approved by Mississippi State University’s International Animal Care and Use Committee (Protocol Number: 14–100), and followed the guidelines set forth by the American Society of Mammalogists [30].

We used a stepwise algorithmic cleaning procedure in R [31] to detect and remove aberrant geographic coordinates from feral pig relocation data. Feral pigs generally travel at speeds of 5 km/hour and have been documented sprinting over short durations up to 50 km/hour [22], thus if the average velocity between consecutive fixes exceeded an average velocity of 20 km/hr, we removed the later location from the dataset on the basis that such excessive speeds could not be realistically maintained over that period and the removed location was likely an error. We additionally discarded all fixes within 24 hours of collar deployment and mortality signals.

Multiple pigs were captured on adjoining properties making it possible for multiple collars to have been deployed within a single sounder. We assessed animal independence by comparing GPS coordinates among all individuals across the same temporal period. We binned time stamps into even hour fixes (e.g., 0200, 0400, 0600, etc.) and only included fixes taken within five minutes surrounding the hour to ensure a consistent temporal window for analysis. We calculated Pearson’s correlation coefficients among all individuals for both latitudinal and longitudinal coordinates. Any pair of individuals with a correlation coefficient |*r|* > 0.5 for both latitude and longitude were considered dependent and one individual from the pair was randomly removed from the analysis. Of the 16 animals that were collared (range of fixes per animal: 1,269–3,417; average fixes per animal: 2,312), 3 were removed due to correlated movements.

### Seasonality

We defined the temporal extent for our analyses based on crop phenology to assess how feral pigs responded to changes in agricultural resource availability. The seasons followed corn phenology because corn is one of the earliest planted crops in the region and constitutes some of the greatest economical losses in the region [32]. In addition, when corn is an available resource, it generally comprises large amounts of feral pig diets in agroecosystems [33]. In similar landscapes (e.g., Louisiana) feral pigs generate some of the greatest economic losses to corn [34] and we wanted to capture the entire phenology following economically valuable crops. We thus defined our seasons as follows: early growing (1 March 2016–15 May 2016); late growing (16 May 2016–31 July 2016); harvest (1 August 2016–31 October 2016); and fallow (1 November 2016–31 January 2017).

### Resource selection

Resource selection analyses describe relative selection for resources based on use in relation to all available resources on the landscape [35]. Not all areas of a landscape are equally available to an animal, therefore the definition of available locations must reflect natural limitations in space use by an animal [36]. To generate locations potentially available to an individual, we sampled randomly across each individual’s 100% minimum convex polygon (MCP; S1 Fig) within each season by generating one available location for every recorded location; this created distinct datasets of availability based on realized home ranges within seasons. We created 100 m buffers around used and available locations and calculated proportional coverage by each land-cover classification to assess relative selection strength. Proportional coverage data provides a more complete window into not only the characteristics of the location of an animal fix, but the surrounding environs as well [6,37].

We fit our resource selection models using generalized linear mixed models following a binomial distribution of sampling error for each season using the *lme4* package [38]. We included the individual as a random intercept to account for individual differences in habitat preference and differing number of locations among individuals [39,40]. Explanatory landscape variables included land cover and distance to the nearest flowline (i.e., streams, canals, ditches; [41]). Agricultural land-cover classifications were obtained from the USDA National Agricultural Statistics Service Cropland Data Layer (CDL) with a 30 m^2^ resolution [42]. We aggregated CDL designations into the following categories: corn, rice, soybean, idle cropland (the reference category), wetland (both woody and herbaceous), other crop (including cotton, sorghum, winter wheat, winter wheat/soy, peas, sod/grass seed, pecan, soybean/oat), and other non-crop (barren, mixed forest, shrub land, grassland, and all developed cover types). We determined these aggregations using model competition with 4 biologically relevant, *a priori* hypotheses based on competing mechanisms of plausible perceived value of different land-cover types by pigs (Table 1). Akaike’s Information Criterion (AIC) consistently ranked the aforementioned aggregations as the top competing model for all seasons (i.e., **Δ**AIC ≥ 2), thus we used these aggregations for all subsequent analyses. Further, proportional coverage data are by necessity collinear; however, the variance inflation factors (VIFs) for all variables (including proportional data and distance to flowlines) were below the recommended threshold value of 3 (VIF < 2 for all coefficients), suggesting negligible multicollinearity in our predictor variables [43].

**Table 1:** Model competition.

Crop Type	Crop Number	Hypothesis 1 Landscape use is determined by both the agricultural and the natural landscape	Hypothesis 2 Landscape use only based on agriculture and the natural landscape is treated equivalently	Hypothesis 3 All agriculture is treated as equivalent and landscape use is determined by the natural landscape	Hypothesis 4 All crops are treated equivalent and all natural landscape is treated as equivalent
Corn	1	Corn	Corn	Crop	Crop
Cotton	2	Other Crop	Other Crop	Crop	Crop
Rice	3	Rice	Rice	Crop	Crop
Sorghum*	4	Other Crop	Other Crop	Crop	Crop
Soybean	5	Soybeans	Soybeans	Crop	Crop
Winter Wheat*	24	Other Crop	Other Crop	Crop	Crop
Winter Wheat/Soybean*	26	Other Crop	Other Crop	Crop	Crop
Peas*	53	Other Crop	Other Crop	Crop	Crop
Sod/Grass Seed*	59	Other Crop	Other Crop	Crop	Crop
Fallow	61	Fallow	Fallow	Crop	Crop
Pecan*	74	Other Crop	Other Crop	Crop	Crop
Aquaculture	92	Water	Water	Water	Water
Water	111	Water	Water	Water	Water
Developed	121–124	Other Noncrop	Other Noncrop	Other Noncrop	Natural
Barren*	131	Other Noncrop	Other Noncrop	Other Noncrop	Natural
Mixed Forest*	143	Other Noncrop	Other Noncrop	Other Noncrop	Natural
Shrubland*	152	Other Noncrop	Other Noncrop	Other Noncrop	Natural
Grassland*	176	Other Noncrop	Other Noncrop	Other Noncrop	Natural
Wetland	190/195	Wetland	Other Noncrop	Wetland	Natural
Double Crop Soybean/Oat*	240	Other Crop	Other Crop	Crop	Crop

Four alternative hypotheses tested to determine the highest-ranking model using Akaike’s Information Criteria (AIC) for determining crop aggregations for resource selection models.

A * indicates that inadequate data for that category existed for each individual, and therefore required binning with another classification. Hypothesis 1 was most favored (i.e., ΔAIC > 2).

We calculated distance to the nearest flowline (i.e., potential water sources) for every location. However, only including linear effects for environmental variables can miss biologically meaningful interpretations, where a quadratic effect can detect an intermediate level at which a species responds most strongly to the variable [44]. We included both linear and quadratic effects for distance to the nearest flowline. Including both linear and quadratic effects can violate the assumption of independence by introducing collinearity; we centered the quadratic effect before squaring, which allows for independent interpretation of the linear term [45,46]. We standardized distance to flowline and the quadratic effect, which enables more direct comparisons among regression coefficients [46,47]. We evaluated statistical significance of model covariates at *α* = 0.05.

Model coefficients within the logistic regression framework may not fully describe the observed patterns of space use [48], particularly for proportional coverage data. It is important to observe that selection for a hypothetical location with 50% cover by a crop type must also include selection for the remaining 50% coverage [6]. We thus calculated the relative probability of selection for a given crop type (corn, rice, and soybean) provided that it co-occurred only with wetlands (the most abundant natural land-cover class in the region)–that is, for a location with proportional coverage *P* by a crop, the proportional coverage by wetlands is 1-*P*. From this perspective, the relative probability of selection *p* for a given location *l* may be calculated as:

$$p(l)=\frac{e^{\beta_{0}+\beta_{c}x_{c}+\beta_{w}x_{w}+\beta_{f,l}x_{f}+\beta_{f,q}x_{f}^{2}}}{1+e^{\beta_{0}+\beta_{c}x_{c}+\beta_{w}x_{w}+\beta_{f,l}x_{f}+\beta_{f,q}x_{f}^{2}}},$$ Eq 1

where *β*_0_ is the intercept; *β*_*c*_, *ß*_*w*_, *ß*_*f*,*l*_, and *ß*_*f*,*q*_ are the seasonal selection coefficients for the crop type, wetlands, and linear and squared (quadratic) distance to flowline, respectively; and *x*_*c*_, *x*_*w*_, and *x*_*f*_ the proportional cover by the crop type, wetlands and the distance to flowline, respectively. Although we fit these as a mixed effects model with random intercepts, the notation in Eq 1 excluded the random effect of the individual in favor of the fixed effects of the general population trends. We plotted these calculated probabilities across the range of possible proportional coverages and include the linear and quadratic effects for distance to flowline, to assess how variation in resource availability influenced selection across seasons. Note that this calculation is not the true probability because we cannot calculate true probability from used/available data fitted to mixed-effect generalized linear models (see [49,50]); these should be interpreted as the relative probabilities of selection within a season.

Lastly, we used the resource selection models to generate maps of the relative probability of selection for any given location within the entire region for each agricultural season using the entire CDL and flowline data for the LMAV. We calculated proportional cover by all land-cover and crop types within a 100 m buffer for each cell of the CDL raster, and calculated distance to flowline from the center of each cell. We used the model predictions to create seasonal maps of the predicted relative probability of feral pig occurrence. Using mixed-effects models accommodates small sample sizes when extrapolating selection patterns to larger spatial extents by reducing individual bias [51]. We also used a novel metric to conduct pairwise comparisons of rasters using a simple metric of raster similarity,

$$S=1-\sqrt{\frac{\sum_{i=1}^{m}\sum_{j=1}^{n}d_{i,j}^{2}}{\sum_{i=1}^{m}\sum_{j=1}^{n}\Delta_{i,j}^{2}}}.$$ Eq 2

Here we defined two *m* x *n* rasters (matrices) **A** and **B** of equal dimension and resolution for which we seek a comparison (e.g., in one possible comparison **A** = the early season raster and **B** = the late season raster). The value *d*_*i*,*j*_ is the difference between cell [*i*,*j*] in **A** and **B**. We also defined a hypothetical raster **α** such that (1) **α** consists only of values of 0 and 1 (the open interval for probability values), and (2) values of 0 and 1 are arranged so as to maximize the difference between **α** and the target raster **B**. The value Δ_*i*,*j*_ is the difference between cell [*i*,*j*] and represents the maximum theoretical difference that could occur if **A** = **α**. The metric *S* is simply the *l*^2^-normalized difference between rasters from 0 (perfect dissimilarity) to 1 (perfect similarity) and may be interpreted as the percent similarity between rasters. This approach allowed us to mathematically quantify the degree of similarity between rasters without requiring statistical assumptions or hypothesis tests that are heavily influenced by sample size.

## Results

Out of 16 feral pigs originally fitted with GPS collars, 13 provided viable data once non-independent animals were removed (4 females and 9 males). Only animals with relocation data that spanned an entire season were included in seasonal analyses which resulted in the following sample sizes: early growing (*n* = 8; $\bar{x}$ = 1,606 fixes/individual; range = 1,436–1,820 fixes), late growing (*n* = 12; $\bar{x}$ = 1,262 fixes/individual; range = 658–1,846 fixes), harvest (*n* = 11; $\bar{x}$ = 1,460 fixes/individual; range = 688–2,178 fixes), and fallow (*n* = 8; $\bar{x}$ = 1,811 fixes/individual; range = 1,304–2,198 fixes).

### Resource selection

**Fig 2:**
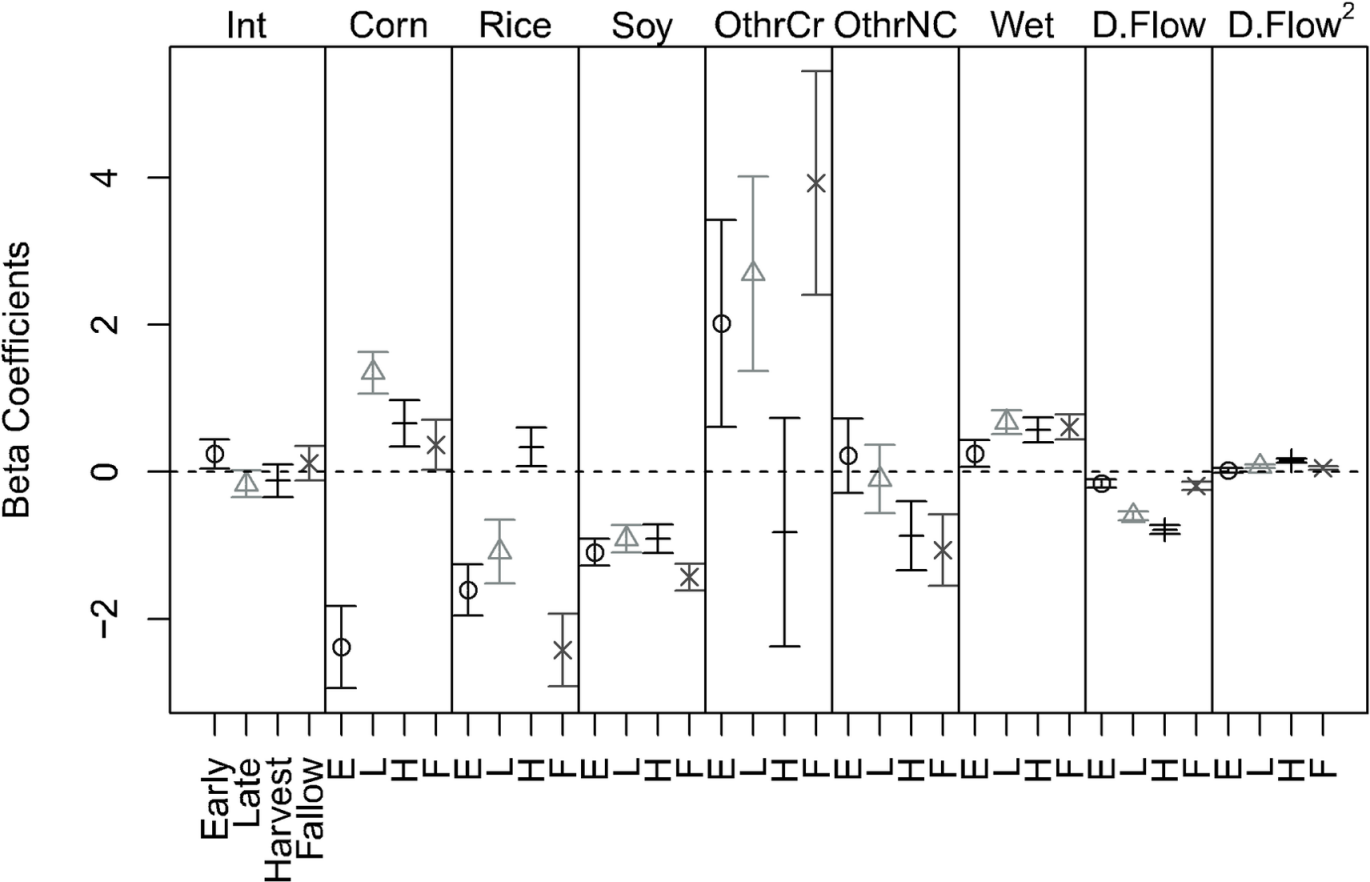
Resource selection beta coefficients. Estimated resource selection for all of the environmental covariates across the four agricultural seasons and their corresponding 95% confidence intervals. Int: intercept; Corn: corn; Rice: rice; Soy: soybean; OtherCr: other crop (i.e., cotton, sorghum, winter wheat, winter wheat/soy, peas, sod/grass seed, pecan, soybean/oat); OtherNC: other non-crop (i.e., barren, mixed forest, shrub land, grassland, and all developed categories); Wet: wetland; D.Flow: linear effect for distance to flowline; D.Flow^2^: quadratic effect for distance to flowline.

Resource selection for habitat covariates changed throughout seasons. Selection for corn was seasonally dynamic, changing from negative in the early season to positive in the late growing season, and decreasing in the harvest and fallow seasons (Fig 2). Selection for rice became positive in the harvest season, indicating increased use of rice relative to the other agricultural seasons (Fig 2). Pigs exhibited positive and relatively constant selection for wetlands throughout all seasons. The “other crop” category had positive selection in all except the harvest season, where selection was neutral (i.e., *β* was not significantly different from 0; Fig 2, S2 Table). The distance to the nearest flowline term represents relative distance away from the location, therefore a negative *ß*-coefficient indicates positive selection for the term in the model output. Selection for distance to flowline increased as seasons progressed and decreased in the fallow season, and pigs demonstrated a significant quadratic effect for distance to flowline in all except the early growing season (Fig 2, S2 Table).

### Selection probabilities

**Fig 3:**
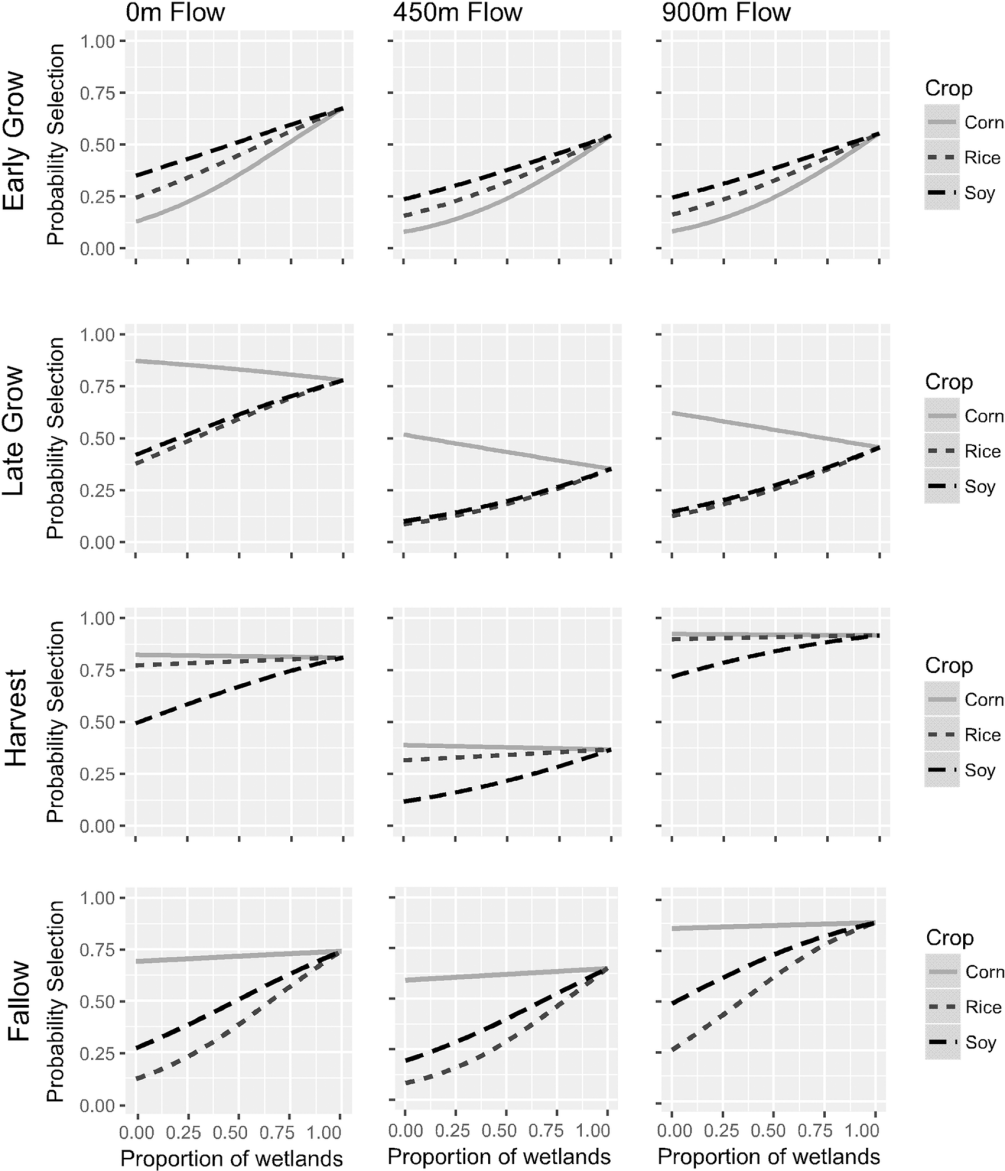
Selection probabilities. Probabilities of use of potential habitat by feral pigs for the major primary resources (i.e. corn, rice and soybean) as a function of proportional coverage by wetlands. Each line corresponds to (1-proportion of wetlands) coverage by a given crop type. Distances represent the proximity of an established flowline.

Several of our model outputs were contrary to our expectations, sometimes dramatically so. For example, we predicted that selection for corn would be strongly positive in the early growing season, yet the *ß*-coefficient for corn in this season was strongly negative, suggesting avoidance (Fig 2). We hypothesized that this deviation from expectation was driven by the dependencies among our predictor variables (i.e., proportion data). We observed selection for such locations during the early growing season was low (i.e., *p*(*l*) < 0.5) when corn, soybean, and rice were in high abundance relative to wetlands (i.e., when *P* = 1; Fig 3). As the landscape included greater abundance of wetlands, and therefore lesser crop abundances, selection increased for these hypothetical locations with the greatest probabilities of selection occurring at 100% coverage by wetlands.

As with our model coefficients, plots of selection for hypothetical locations were inconsistent across seasons. For example, we found in the early growing season both rice and soybean had higher selection probabilities than corn, whereas corn became the highest selected crop from the late growing season through the harvest season (Fig 3). However, in the late growing season the relative probability of selection for corn decreased as corn was in closer proximity to wetlands at all distances from an established flowline. The relative probability of selection for crops followed similar trends in the late growing season and harvest season, with the exception of increased probability of selection for rice in the harvest season relative to the other seasons (Fig 3). In the fallow season, corn had the highest relative probability of selection while rice and soybean remained low until greater proportional coverage by wetlands occurred (Fig 3).

Further, selection probabilities varied across distances to flowlines. Our model outputs indicated a significant quadratic effect for distance to flowline, so we modeled how selection probabilities changed at varying distance to flowlines for distances at the 0%, 50%, and 100% quantiles (i.e., 0 m, 450 m and 900 m). Some general trends emerged when modeling a quadratic effect for distance to flowline. The lowest selection probabilities for corn, rice, and soybeans consistently occurred when feral pigs were located 450 m from an established flowline except in the early growing season, when no significant quadratic effect was observed (Fig 3).

### Predicted probability of use

**Fig 4:**
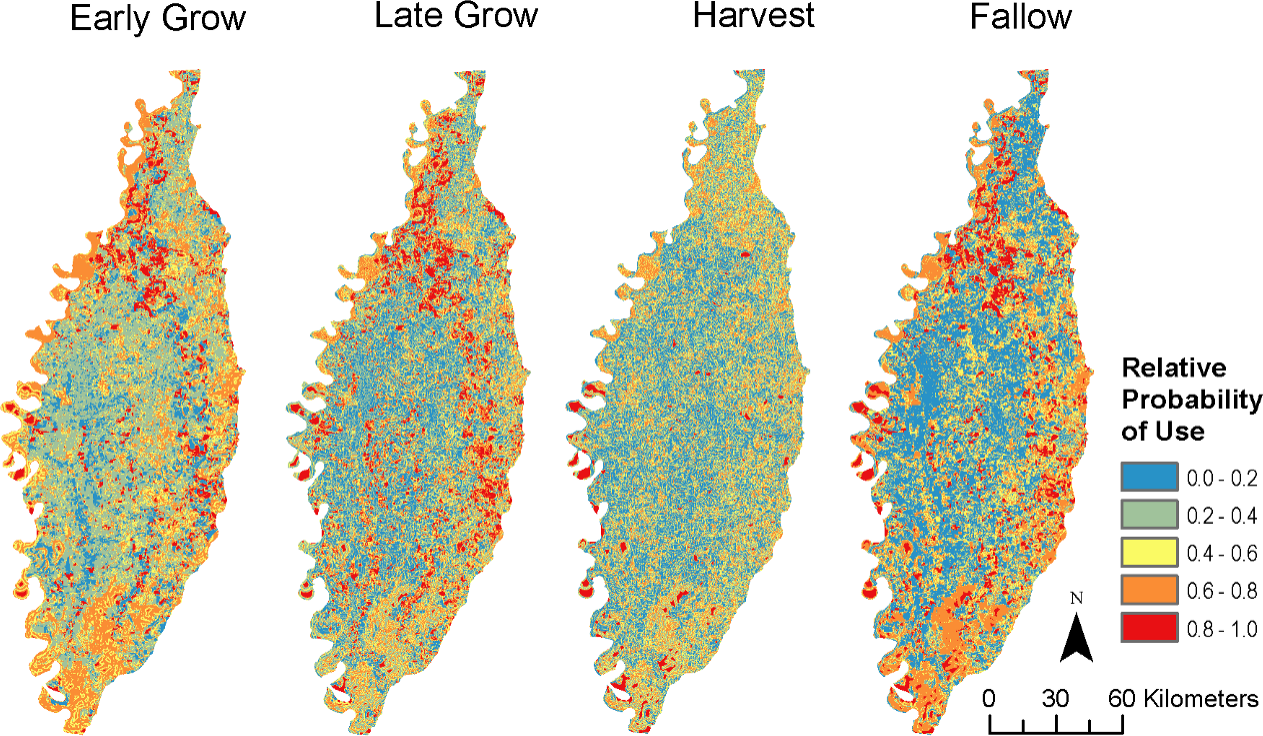
Predicted probabilities of use. Predicted probabilities of feral pig resource selection across the Mississippi Alluvial Valley for the defined agricultural seasons.

To better understand the realized implications of our models for space use, we solved each seasonal model across the LMAV and produced heatmaps from the probabilities of selection per pixel (Fig 4). The heatmaps indicate regions with high probabilities of selection and demonstrate strong seasonal variation in the likely distributions of pigs. Changes among seasons showed marked differences, particularly in relation to the harvest season where relative probability of selection tended to decline toward spatial uniformity (Fig 4). Although the maps appear visually similar, our similarity metric indicated the maps represent different temporal patterns of space use by feral pigs, with some pairwise comparisons only producing ~60% similarity (Table 2).

**Table 2:** Raster comparison.

	Early Grow	Late Grow	Harvest	Fallow
**Early Grow**	1.0000000			
**Late Grow**	0.6860769	1.0000000		
**Harvest**	0.6170393	0.7281223	1.0000000	
**Fallow**	0.7188552	0.8114958	0.6612455	1.0000000

Comparison between the predictive maps to establish differences between seasons. Larger values indicate stronger similarities between predictive maps.

## Discussion

We found strong evidence of season-specific differences in resource selection driven by the seasonal availability of agricultural resources. Not only did overall selection strength for proportional cover across the landscape vary (Fig 2), but the relative probability of selecting the primary agricultural crops (i.e., corn, rice, and soybeans) changed throughout seasons as well (Fig 3). Even with relatively small sample sizes, we were able to detect changes in the spatial distribution patterns of feral pigs. The marked changes in resource selection throughout seasons indicate that feral pigs respond to vegetative phenology which varies in nutritional quality and abundance throughout maturation, supporting our hypothesis. However, our prediction that corn would be strongly selected in the early growing season was only demonstrated through the relative probability of selection in strongly mixed habitat (i.e., corn co-occurs with natural wetlands; Fig 3), which is likely due to the lack of anti-predatory cover available in the early growing stages of development. Human hunting pressures in the LMAV occur year-round [29], requiring reliable sources of cover which can change seasonally based on crop phenology. These results suggest feral pigs exhibit changing behavioral preferences driven by changes in how and why they use corn, consistent with other findings. For example, Podgórski et al. (2013) found that feral pigs have short foraging bouts in human-dominated landscapes when relatively little cover is available. This would naturally result in selection for forage resources being dependent on an availability gradient of human settlements and development (consistent with the habitat functional response; [14]).

The habitat functional response can also be driven by thermoregulatory considerations, as has been demonstrated in other ungulates [6], and this seems a likely driver for pigs as well given their use of muddy wallows to thermoregulate [22]. This helps explain why crops generally exhibit higher selection probabilities when close to wetlands; foraging from high quality crop resources while in proximity of high quality thermoregulatory habitat should increase individual fitness via increased foraging efficiency [23] and thermoregulatory efficiency [52]. Bottomland hardwoods also provide antipredatory cover and seasonal fluxes of high quality forage (e.g., hard and soft mast), which should also affect the functional response in risky locations [53]. As such, at least seasonally, crop consumption by pigs may be viewed as a form of facultative foraging [54] occurring in response to preferential selection for thermoregulatory and seasonal foraging habitat, reinforced by antipredatory benefits of natural cover.

It is important to note that the “other crop” category has high selection throughout all but the harvest season (Fig 3). This category includes numerous food crops including pecans, peas, sorghum, and double-cropped fields where multiple crops are planted in the same field in a given year to maximize land production. Double-cropping seems particularly relevant here because it provides alternative food sources within the early growing and fallow seasons (usually as winter wheat, *Triticum aestivum*). Wetlands were consistently selected for in each season but less strongly in the early growing season which coincides with increased resource availability in double-cropped fields and seasonal flooding which increases waste grain availability [55]. Spring flooding in bottomland hardwoods can also make resources seasonally inaccessible and alter resource availability. Wetlands provide hard and soft mast as forage at different times of year [12], and low mast availabilities would drive feral pigs toward alternative resources, such as winter wheat. Additional investigation can refine the influences of double-cropped fields on space use; however, our data were insufficient to make such distinctions.

Our results underscore the importance of the composition of the landscape for wildlife movements and space use. Selection for locations varying in proportional cover between crops and natural wetlands was highly dependent on the relative proportions of each, with a general trend that increasing proportionality by wetlands increases the likelihood of selection (Fig 3). This implies selection by pigs for crops is driven more by wetland availabilities and their proximity to croplands than by the crops themselves (reinforcing the notion of facultative foraging), which in the LMAV would constitute mostly bottomland hardwood forests within 100 m from a crop (the radius of our buffers). Within the context of the habitat functional response, given that pigs are generalist omnivores, it seems likely their space use would be driven more by thermoregulatory considerations than by foraging; indeed, most croplands were avoided in all seasons when in high abundance relative to wetlands (Fig 3). If so, then damage by pigs to agricultural interests is largely context-dependent. The animals may not spend a lot of time in particular crop fields, but should they find themselves in an area with readily available crops, they will utilize them. This behavior is consistent with opportunistic foraging [12,17] and suggests current trapping procedures for pig management could be improved by better understanding the interactions among landscape structure, proximity, and preferential selection by pigs. However, the direction and magnitude of the effect of proportional coverage was strongly influenced by the season (Figs 2 and 3) and realized patterns of space use among seasons was inconsistent (Fig 4, Table 2). As such, the habitat functional response for thermoregulatory habitat is likely also season-specific.

Our findings suggest feral pigs change their space use trends by altering the relative probability of selection for the primary crops. In the early growing season corn is one of first resources planted, but rice and soybeans have higher probabilities of selection during the early growing season (Fig 4). This could be due to the rapid development of corn whereby the protein-rich seed germinates within a few days under ideal conditions [15] into a less palatable plant. Corn becomes the strongest selected crop within all other seasons (Fig 3). Corn maturation decreases the sugar and crude proteins available [56], rendering the crop less energetically beneficial as a food item but perhaps increasing its value as a source of cover throughout maturation (e.g., late growing season). This partly explains the higher estimated selection for corn in the late growing and harvest seasons, though we emphasize that wetlands remain the most likely source of thermoregulatory cover for pigs. During the fallow season, the selection probabilities for all crops are high despite the season suggesting the landscape is barren. However, corn, rice, and soybean produce the largest amount of waste grain within the LMAV [27], which as noted above increases available forage. Additionally, the high selection probabilities could be attributed to landowners that manage for other wildlife species, such as white-tailed deer and waterfowl, which supplies additional forage through feeders or winter crops that may otherwise be unavailable [57].

We detected a significant effect of distance to flowlines (Figs 2 and 3), which yields two critical conclusions. First, this reinforces the notion that the habitat functional response is largely driven by the availability of wetlands (i.e., for thermoregulation). For example, in the early growing season all crop types had a high relative probability of selection at low abundance relative to wetlands (Fig 3), consistent with the functional response as described above, but the overall magnitude of selection for such locations declined at intermediate distances from a flowline (i.e., 450 m). This may suggest many water sources at this distance are ephemeral and only temporarily wet following rainfall, resulting in an overall lower preference than for permanent wetlands; however, our data are insufficient to examine this possibility. Second, in most seasons selection for the maximum distance from flowline increased to levels observed at the minimum distance to flowline. Given our general findings in favor of selection for wetlands, we expect this phenomenon likely corresponds to selection for water sources not present in the flowline dataset (e.g., permanent wetlands, irrigation). If such is the case, it indicates that within the typical seasonal use area, animals in this system need travel no more than ~450 m to find water of some type, and we have gained information about the animal’s range from its behavior alone. This is an important conclusion suggesting that radio-tracked animals may be used as “remote sensors” of environmental quality and landscape structure. This is similar in concept to the notion of indicator species whereby presence or absence of the organism may suggest quality or health of a particular location [58], except that it is now the explicit behaviors and movements of individuals informing both the quality of locations they encounter, and the general structure of their occupied range. This could be a powerful tool for supplementing classical remote sensing techniques to improve remote assessment of landscape composition and configuration.

We expected decreased selection strength for corn at later stages of maturity when other crops become more nutritionally valuable relative to corn [7,15]; however, we observed increased selection for corn during seasons corresponding to plant maturity (Figs 2 and 3). We do not suspect this increased selection strength is driven exclusively by the increased availability of corn at later maturity because other crops (e.g., rice, soybean) are as easy to apprehend and also readily available across the landscape (Fig 1). Rather, we expect increased selection to be driven by the value of corn as cover in the late growing when mean daytime temperatures are higher—that is, the animals change the way they interact with the resource, treating it more as shelter rather than forage while corn grain matures [33]. Agricultural crops comprise the majority of forage when seasonally available relative to mast items [12,33], and during the late growing season corn can be used as an additional source of cover to facilitate access to more preferred resources. This sort of “use-switching” has significant implications for plant/animal interactions in general. Consider observed changes in plant phenology in response to climatic variations [59,60]. This has resulted in changes in species distributions and competitive interactions between plants and animals, with the specific mechanism underlying these changes being dependent on biotic interactions [61]. Such effects of plant phenology on animals have been observed as suppressed nutrition resulting in changes in the timing of birth and demography of roe deer (*Capreolus capreolus*) [62], and in reduced recruitment in Shiras moose (*Alces alces shirasi*) [63]. In pigs, we observed behavior consistent with use-switching to facilitate use of shelter, suggesting that use-switching interacting with plant phenology is at least one mechanism by which climate indirectly influences animal populations.

Proportional coverage data are common in habitat selection studies from ungulates [6,64] to avifauna [65]. Our study highlights the difficulties in interpreting models fit to such data. Resource-selection model outputs using proportional coverage data represent selection for a particular location based on the relative abundance of resources rather than selection for a specific resource type, as these outputs are commonly interpreted [66,67]. The aggregation of land-cover classifications into proportional cover may thus produce *ß*-estimates that are nonsensical when viewed in isolation, as we observed here (Fig 2), therefore only interpreting the *ß*-coefficients can be misleading without consideration of the realized theoretical and spatial patterns predicted by the full model. Our work stresses the importance of proper interpretation and consideration of specific model outputs using graphical approaches, particularly for proportion data, rather than simply relying on model coefficients and statistical significance.

We found that a generalist omnivore responds to agricultural phenology via a functional response in resource selection. This suggests that although generalist organisms can survive in a wide array of environmental conditions, they maintain distinct selection preferences in the resources they exploit, and these preferences are likely inconsistent among agricultural seasons. The temporal availability of agricultural resources leads to changes in spatial distributions, which can be incorporated into species management by considering crop phenology and critical spatiotemporal scales and extents. For an invasive species such as feral pigs, the overall patterns and predictive maps can be used to inform management decisions for population control. Our findings particularly suggest a habitat functional response in an agricultural landscape not only could, but indeed should, exhibit temporal inconsistencies based on crop phenologies and abiotic conditions, and that no “one size fits all” management approach is likely to exist for wildlife in agricultural systems, nor for the landscapes they occupy. Future studies should consider the system- and species-specific ecological mechanisms driving observed changes in resource selection that ultimately determine an organisms’ distribution, particularly beyond the single-year design used here to examine the consistency of these findings across broad temporal extents.

## Conclusions

We examined how a landscape characterized by strong seasonal differences in resource quality and quantity evokes temporal variation in animal behavior, and in resulting animal distributions. We demonstrated season-specific preference for resources by feral pigs in an agricultural landscape, driven by seasonal availability and plant phenology, and identified a likely season-specific functional response in resource selection. We also identified a possible behavioral link between plant phenology and animal distributions (i.e., “use-switching”). Our results indicate the importance of studying animal responses to season-specific landscape composition as it can affect their movements and overall space use patterns, leading to season-specific mechanisms influencing a single ecological phenomenon. We also stress the importance of proper model interpretation and prediction from complex or strongly collinear data, particularly proportional coverage data. Acknowledging such collinearity can aid in identifying interesting ecological phenomena (e.g., how two collinear variables change together according to a particular model), and failure to do so can lead to erroneous or misleading conclusions regarding a species’ ecology. Our work highlights the importance of carefully considering seasonality in animal preference and provides guidance on fitting models relevant to such studies, particularly in understudied regions for wildlife studies (i.e., agricultural systems).

## Supporting information

S1 Table Landscape composition. Percent cover by each CropScape land cover classification across the Lower Mississippi Alluvial Valley.
(PDF)

S2 Table Beta estimates for seasonal resource selection models. Model outputs from generalized linear mixed effect models of feral pig resource selection throughout agriculturally defined seasons during the day. A bolded value represents a significant coefficient.
(PDF)

S1 Fig Sampling seasonal availability. Seasonal 100% minimum convex polygons used to sample availability for each individual in the late growing season to show the overall extent of the Lower Mississippi Alluvial Valley sampled.
(PDF)
