# Supplementary material for: Seasonal variation in preference dictates space use in an invasive generalist
Source: PLoS One. 2018 Jul 20;13(7):e0199078. doi: 10.1371/journal.pone.0199078 (PMC6054371; doi:10.1371/journal.pone.0199078)
Supplement: S1 Table — Percent cover by each CropScape land cover classification across the Lower Mississippi Alluvial Valley. (PDF) [file pone.0199078.s001.pdf]

S1 Table. Landscape composition. Percent cover by each CropScape land cover classification across the Lower Mississippi Alluvial Valley.

CDL Value	*Crop Type*	*Pixel Count*	*Area sq. km*	*Percent Cover*
1	Corn	2452461	2207.2149	11.9
2	Cotton	1285627	1157.0643	6.24
3	Rice	785099	706.5891	3.81
4	Sorghum	14885	13.3965	0.07
5	Soybeans	7784781	7006.3029	37.77
6	Sunflower	85	0.0765	0.00
10	Peanuts	36998	33.2982	0.18
24	Winter Wheat	32074	28.8666	0.16
26	Dbl Crop WinWht/Soybeans	91283	82.1547	0.44
27	Rye	7	0.0063	0.00
28	Oats	1137	1.0233	0.01
29	Millet	289	0.2601	0.00
37	Other Hay/Non Alfalfa	7495	6.7455	0.04
44	Other Crops	13	0.0117	0.00
46	Sweet Potatoes	11770	10.593	0.06
48	Watermelons	3	0.0027	0.00

53	Peas	659	0.5931	0.00
59	Sod/Grass Seed	9313	8.3817	0.05
61	Fallow/Idle Cropland	1416281	1274.6529	6.87
74	Pecans	38790	34.911	0.19
92	Aquaculture	10186	9.1674	0.05
111	Open Water	935344	841.8096	4.54
121	Developed/Open Space	631837	568.6533	3.07
122	Developed/Low Intensity	105219	94.6971	0.51
123	Developed/Med Intensity	68448	61.6032	0.33
124	Developed/High Intensity	15174	13.6566	0.07
131	Barren	22081	19.8729	0.11
141	Deciduous Forest	2768	2.4912	0.01
142	Evergreen Forest	1493	1.3437	0.01
143	Mixed Forest	626	0.5634	0.00
152	Shrubland	11130	10.017	0.05
176	Grassland/Pasture	11483	10.3347	0.06
190	Woody Wetlands	4798175	4318.3575	23.28
195	Herbaceous Wetlands	20040	18.036	0.1

205	Triticale	1230	1.107	0.01
222	Squash	3	0.0027	0.00
225	Dbl Crop WinWht/Corn	4630	4.167	0.02
229	Pumpkins	2	0.0018	0.00
240	Dbl Crop Soybeans/Oats	2695	2.4255	0.01
