# Supplementary material for: Seasonal variation in preference dictates space use in an invasive generalist
Source: PLoS One. 2018 Jul 20;13(7):e0199078. doi: 10.1371/journal.pone.0199078 (PMC6054371; doi:10.1371/journal.pone.0199078)
Supplement: S2 Table — Model outputs from generalized linear mixed effect models of feral pig resource selection throughout agriculturally defined seasons during the day. A bolded value represents a significant coefficient. (PDF) [file pone.0199078.s002.pdf]

Supporting information

S2 Table. Beta estimates for resource selection models. Model outputs from generalized linear mixed effect models of feral pig resource selection throughout agriculturally defined seasons during the day. A bolded value represents a significant coefficient.

	**Early Growing**			**Late Growing**			**Harvest**			**Fallow**		
	Estimate	*Std. Error*	*p-value*	*Estimate*	*Std. Error*	*p-value*	*Estimate*	*Std. Error*	*p-value*	*Estimate*	*Std. Error*	*p-value*
**Fixed Parts**												
Intercept	0.52	0.19	**0.005**	-0.10	0.11	0.39	-0.14	0.12	0.242	0.60	0.16	**<.001**
Corn	-7.27	1.65	**<.001**	1.22	0.20	**<.001**	0.19	0.27	0.495	0.23	0.33	0.49
Rice	-3.52	0.36	**<.001**	-2.14	0.36	**<.001**	0.26	0.16	0.117	-5.11	0.57	**<.001**
Soybean	-2.98	0.15	**<.001**	-1.98	0.14	**<.001**	-1.15	0.13	**<.001**	-2.74	0.13	**<.001**
Other Crop	-5.23	1.54	**<.001**	-1.12	1.10	0.313	-3.26	1.13	**0.004**	-0.91	1.01	0.37
Other Noncrop	-1.26	0.30	**<.001**	0.02	0.28	0.938	-1.08	0.29	**<.001**	-2.73	0.30	**<.001**
Wetland	0.36	0.10	**<.001**	0.81	0.09	**<.001**	0.61	0.10	**<.001**	0.07	0.10	0.45
Dist Flow	-0.15	0.03	**<.001**	-0.76	0.04	**<.001**	-1.01	0.04	**<.001**	-0.38	0.04	**<.001**
Quad Flow	-0.01	0.02	0.731	0.11	0.01	**<.001**	0.23	0.02	**<.001**	0.08	0.02	**<.001**
**Random Parts**												
Variance		0.03			0.03			0.06			0.05	
Std. Deviation		0.17			0.18			0.25			0.23	
N_{Pigs}		8			12			11			8	
Observations		6294			7264			7544			6996	
