# Supplementary material for: Seasonal variation in preference dictates space use in an invasive generalist
Source: PLoS One. 2018 Jul 20;13(7):e0199078. doi: 10.1371/journal.pone.0199078 (PMC6054371; doi:10.1371/journal.pone.0199078)

S1 Figure. Seasonal availability sample. Seasonal 100% minimum convex polygons used to sample availability for each individual in the late growing season to show the overall extent of the Lower Mississippi Alluvial Valley sampled.

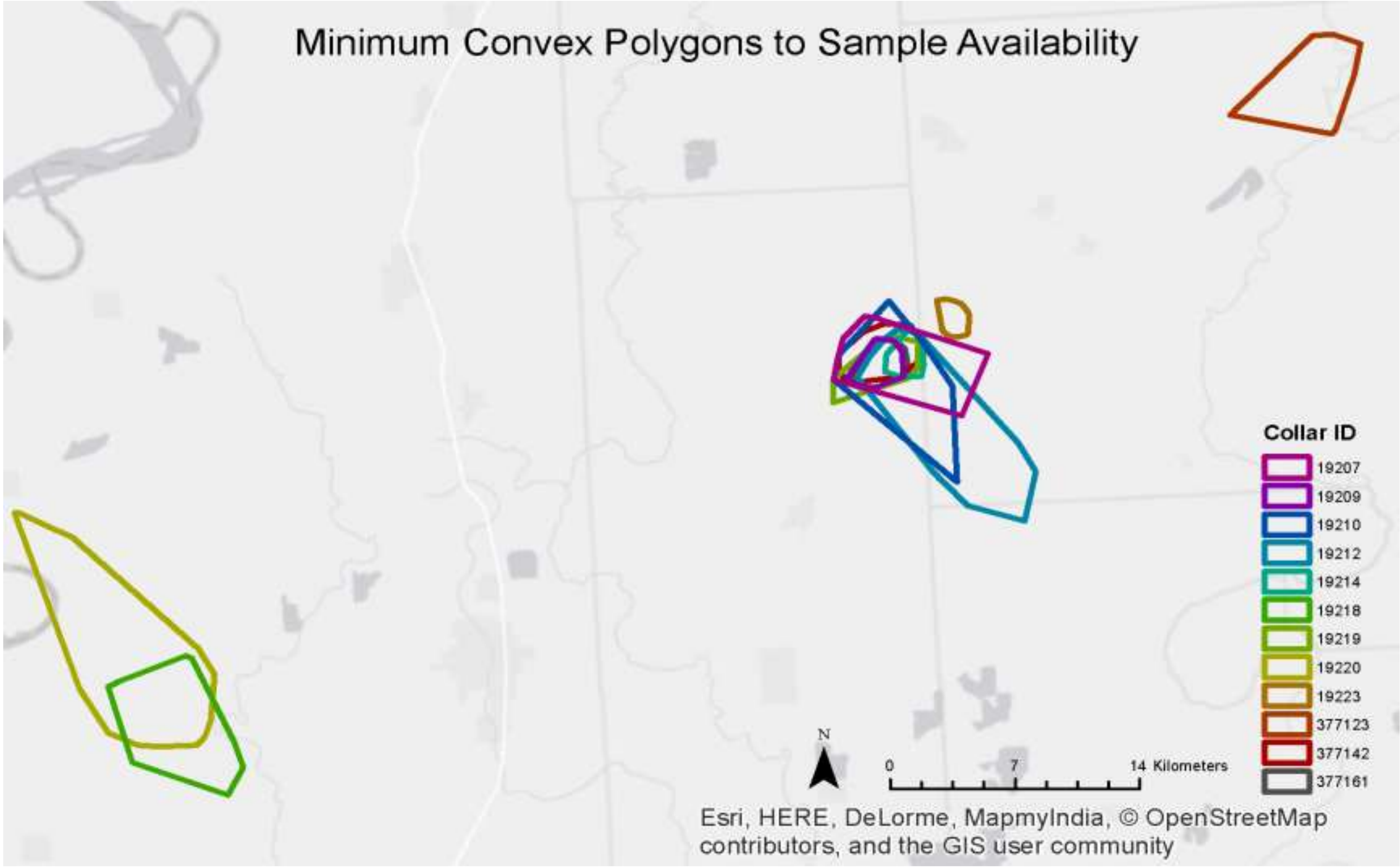

Supplement: S1 Fig — Seasonal 100% minimum convex polygons used to sample availability for each individual in the late growing season to show the overall extent of the Lower Mississippi Alluvial Valley sampled. (PDF) [file pone.0199078.s003.pdf]
